# RETRACTION: An in‐depth analysis of wound incidence and injury status among professional athletes: A comprehensive review

**DOI:** 10.1111/iwj.70130

**Published:** 2024-11-12

## Abstract

**RETRACTION:** HuangX. , WuY. , LiX. , ZhangM. , LiuY. , “An in‐depth analysis of wound incidence and injury status among professional athletes: A comprehensive review,” International Wound Journal 21, no. 4 (2024): e14873, 10.1111/iwj.14873.38629589 PMC11022304

The above article, published online on 23 April 2024, in Wiley Online Library (http://onlinelibrary.wiley.com/), has been retracted by agreement between the journal Editor in Chief, Professor Keith Harding; and John Wiley & Sons, Ltd. Following an investigation by the publisher, the parties have concluded that this article was accepted solely on the basis of a compromised peer review process. Therefore, the article must be retracted. The authors did not respond to the notice of retraction.

**RETRACTION:** X. Huang , Y. Wu , X. Li , M. Zhang , Y. Liu , “An in‐depth analysis of wound incidence and injury status among professional athletes: A comprehensive review,” International Wound Journal 21, no. 4 (2024): e14873, 10.1111/iwj.14873.38629589 PMC11022304

The above article, published online on 23 April 2024, in Wiley Online Library (http://onlinelibrary.wiley.com/), has been retracted by agreement between the journal Editor in Chief, Professor Keith Harding; and John Wiley & Sons, Ltd. Following an investigation by the publisher, the parties have concluded that this article was accepted solely on the basis of a compromised peer review process. Therefore, the article must be retracted. The authors did not respond to the notice of retraction.

